# Insights into intramuscular adipose–muscle signaling in the diabetic lower extremity

**DOI:** 10.1016/j.jcte.2025.100422

**Published:** 2025-10-28

**Authors:** Chang Gui, Dakota R. Kamm, Jeremie L.A. Ferey, Kathryn L. Bohnert, Jeremy J. McCormick, Mary K. Hastings, Gretchen A. Meyer

**Affiliations:** a Department of Biomedical Engineering, Washington University, St. Louis, MO, USA; b Program in Physical Therapy, Washington University, St. Louis, MO, USA; c Department of Orthopaedic Surgery, St. Louis, MO, USA; d Department of Neurology, Washington University, St. Louis, MO, USA

**Keywords:** Intermuscular adipose tissue, Regeneration, Adipose biology, Signaling crosstalk, Lower limb amputation

## Abstract

Intramuscular adipose tissue (IMAT) has been proposed to directly contribute to myofiber dysfunction through paracrine signaling. The impacts of this signaling beyond contributing to myofiber insulin resistance are largely unknown. This study aims to explore the human IMAT transcriptome, with a focus on its potential role in myoblast fusion deficits in advanced muscle pathology. Using a within-subjects design, we compared IMAT to subcutaneous (SQ) fat in individuals with and without diabetes undergoing below-knee amputation. We hypothesized that IMAT from the diabetic group would exhibit a pro-inflammatory profile, similar to diabetic SQ, and that inflammatory secreted factors from IMAT progenitors would impair cultured myoblast fusion. Instead, we found that the IMAT transcriptome from the diabetic group exhibited reduced enrichment of inflammatory pathways compared with SQ and less transcriptional evidence for immune cell infiltration. While IMAT featured a mostly anti-myogenic transcriptional profile for secreted cytokines, media conditioned by IMAT progenitors did not uniquely impair fusion of cultured myoblasts compared with SQ. Surprisingly, the diabetic status of the myoblast donor predicted myoblast fusion, with reduced fusion rates in diabetic myoblasts exposed to conditioned media from all adipose sources. This suggests that IMAT-myoblast signaling may be detrimental to regeneration in diabetes, but that the effect is driven in part by an intrinsic difference in diabetic myoblasts’ sensitivity to IMAT secreted factors. This emphasizes the insight that can be gained from disease-state matched and mismatched culture models and highlights the need to better understand how diabetes impacts myoblasts and their interaction with the disease environment.

## Highlights

• Excess IMAT could impair myoblast differentiation through paracrine signaling.
• Transcriptional differences between IMAT and SQ suggest unique signaling by IMAT.
• Both IMAT and SQ conditioned media impaired diabetic myoblast differentiation.
• Sensitivity of myoblasts to adipokines may drive impaired regeneration in diabetes.

## Graphical abstract

**Insights into Intramuscular Adipose–Muscle Signaling in the Diabetic Lower Extremity.** This study compared biopsies of intramuscular adipose tissue (IMAT) and subcutaneous adipose tissue (SQ) using transcriptional profiling, histological analyses and cell culture models to better understand the effect of IMAT on myogenesis (green arrow).

**Figure:**
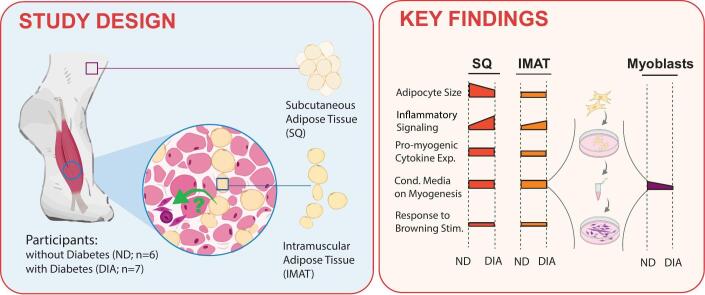

## Introduction

Muscle pathology associated with advanced type 2 diabetes extends beyond insulin resistance to include myofiber atrophy, degeneration, impaired contractility and regenerative deficits, especially in chronic diabetes with co-morbidities [[1], [2], [3]]. These changes are predicted to cumulatively decrease muscle strength and physical function, impacting quality of life [2,4]. Regenerative deficits are particularly impactful in the framework of rehabilitation as muscle progenitor (satellite) cells are key players in muscle plasticity and recovery of function. Remarkably, even with extensive muscle structural pathology, attempted muscle regeneration persists [1], suggesting the potential for therapeutic targeting throughout the pathogenesis of diabetes.

Satellite cell dysfunction has been proposed to be directed by changes in the systemic or local signaling environment [[5], [6], [7]], reviewed in [8]. The mediators have yet to be fully identified, but inflammatory cytokines and adipokines are implicated [5,9]. Limb muscle of individuals with diabetes features a progressive accumulation of intramuscular adipose tissue (IMAT; adipocytes and associated cells between muscle fibers) that is thought to modify the local biophysical and biochemical muscle microenvironment (reviewed in [10]), making it a likely source for anti-myogenic signaling. Indeed, IMAT isolated from individuals with obesity secreted higher levels of inflammatory cytokines with anti-myogenic action compared with subcutaneous (SQ) fat [11]. While the effect of IMAT secreted factors on regeneration has not been explored, SQ adipose conditioned media can reduce myogenesis in cultured human myoblasts [9], an effect supported by animal studies [12,13]. Thus, the closer juxtaposition of IMAT and potentially higher inflammatory cytokine secretion suggests that IMAT secreted factors could heavily influence muscle regeneration.

The dearth of studies on IMAT relative to SQ fat stems primarily from the difficulty in isolating sufficient quantities of it reliably from muscle biopsy [10]. In this study, we utilize large (>10 g) muscle and adipose samples acquired as part of a tissue procurement collective resource from individuals undergoing elective below-knee amputation to obtain volumes of IMAT sufficient for RNA sequencing, histological assessment and progenitor cell isolation for each participant. These samples were subdivided by those with a clinical diagnosis of type 2 diabetes and those without, and IMAT and SQ measures were paired by individual for a within-subjects analysis. Individuals in both groups were sedentary with limited mobility and co-morbidities and represent clinical populations seeking major orthopaedic and rehabilitative care, rather than those with well-managed diabetes and healthy controls. We utilize these samples to provide insight into unique morphology and signaling in IMAT in advanced diabetic muscle pathology and its potential to locally modulate myogenesis and impact muscle regeneration. We hypothesized that the transcriptional profile of IMAT would be unique in those with a diagnosis of diabetes, driving an inflammatory and anti-myogenic secretory profile which would inhibit myogenesis in a conditioned media culture model.

## Materials and methods

### Study approval and design

This study was approved by the Human Research Protection Office at Washington University School of Medicine and all participants gave written informed consent. Participants were selected from patients scheduled for elective below-knee amputation with the Foot & Ankle Service at Washington University School of Medicine and Barnes Jewish Hospital. Participants were divided by clinical diagnosis of type 2 diabetes into diabetic (DIA) and non-diabetic (ND) groups. Samples of abductor hallucis muscle, abductor hallucis IMAT (microdissected from bulk muscle) and SQ adipose tissue from the lower calf were collected during surgery and processed as described for each method, and as previously reported [1]. Subacromial adipose tissue was collected from patients scheduled for elective rotator cuff surgery and processed identically to IMAT and SQ.

### Adipose histopathology

A portion of each adipose sample was fixed in formalin, embedded in paraffin and sectioned at 10 μm on a microtome. Sections were stained with Hematoxylin and Eosin (H&E) to visualize morphology and immunostained to quantify macrophages. Briefly, de-paraffinized and hydrated sections were subjected to antigen retrieval in sodium citrate buffer (10 mM tri-sodium citrate, 0.05 % Tween-20, pH 6.0) for two hours at 57 °C, followed by a graded return to room temperature in deionized water. Sections were then permeabilized for 10 min in 1 % triton X-100, blocked for 1 h in 2.5 % goat serum and incubated overnight in primary antibodies against CD68 (Abcam Ab955; 1:200) and laminin (Abcam Ab11575). Fluorescent secondaries and DAPI were used to visualize CD68+ macrophages, which were identified as CD68+ areas overlapping a DAPI+ nucleus using manual thresholding and a semi-automated ImageJ algorithm. Areas of CD68+ regions of interest and manually-drawn adipocyte regions of interest were computed in ImageJ.

### RNA isolation and sequencing

A portion of each adipose sample was flash-frozen for RNA isolation and sequencing. RNA was extracted via a combination Trizol/chloroform extraction and RNEasy mini kit (Qiagen, Hilden, Germany) with DNAse treatment. Libraries were prepared by the Genome Technology Access Center according to the manufacturer’s protocol, indexed, pooled, and sequenced on an Illumina NovaSeq 6000. Basecalls and demultiplexing were performed with Illumina’s bcl2fastq software and a custom python demultiplexing program. RNAseq reads were then aligned to the Ensembl Release 101 annotations with STAR. Gene counts were derived from the number of uniquely aligned unambiguous reads by Subread:featureCounT. Isoform expression of known Ensembl transcripts were estimated with Salmon. A set of genes highly elevated in skeletal muscle was obtained from the Human Protein Atlas. After filtering out muscle-specific genes, low-count genes with sum of raw counts across all samples < 50 were removed. All remaining protein-coding gene counts were then imported into the R/Bioconductor package DESeq2 Differentially Expressed Gene (DEG) analysis. Within DESeq2, normalized counts were determined by relative log expression (RLE) and DEGs were determined with a false discovery rate (FDR) < 0.05 and absolute log2FC > 2 using within-subjects matching to account for inter-participant variability. Principal component analysis was visualized using ggplot2 package in R. Pathway analysis was performed with gene set enrichment analysis (GSEA) or Kyoto Encyclopedia of Genes and Genomes (KEGG) Mapper. Genes with a secreted signal peptide were identified using SignalP 6.0 [14].

### Adipose progenitor cell isolation and culture

A portion of each adipose sample was digested for adipose progenitor cell (APC) isolation and culture, as previously described [15], and in the Supplemental Methods. Briefly, APCs were dissociated from partially digested tissue, expanded in culture to passage 2–4, grown to confluence and assigned to one of three treatment groups: 1) no differentiation (standard growth media), 2) adipogenic differentiation (adipogenic induction and maintenance media) and 3) adipogenic differentiation and stimulation (10 μM isoproterenol). Following this culture protocol, cells were washed three times with PBS and incubated in low-glucose DMEM for 24 h for conditioning. Conditioned DMEM was used to make myogenic induction media which was applied to myoblast cultures described below. Following DMEM collection, APC cultures were washed with PBS and collected in Trizol for RNA extraction.

### Myoblast isolation and culture

A portion of each muscle sample was digested for myoblast isolation as previously described (1), and in the Supplemental Methods. Briefly, cells were dissociated from partially digested muscle, plated to isolated adherent cells and banked. At the time of experimentation, banked cells were FACS sorted to isolate myoblasts (CD45-/CD31-/CD56+), which were expanded to passage 2, cultured to 70 % confluency and treated with conditioned myogenic induction media (described above). Myoblasts from 2 ND and 2 DIA participants were cultured separately with the same 16 APC conditioned medias (4 ND SQ, 4 DIA SQ, 4 ND IMAT and 4 DIA IMAT), resulting in condition-matched (ND APCs: ND myoblasts, DIA APCs: DIA myoblasts) and condition-mismatched (ND APCs: DIA myoblasts, DIA APCs: ND myoblasts) pairings. Myoblasts treated with unconditioned myogenic induction media served as an internal control for each participant. Following three days in the media, with no media change, half of the myoblast cultures were collected in Trizol for RNA extraction while another half were washed with PBS and fixed with pre-chilled methanol for myosin heavy chain (MHC) immunostaining.

### RT-qPCR

The total RNA of APCs and myoblasts was extracted using phenol–chloroform as previously described [15] and in the Supplemental Methods. cDNA was prepared with MultiScribe reverse transcription kit (Applied Biosystems; 4368814) according to manufacturer’s instructions. Then, qPCR was performed with cDNA and fast SYBR Green PCR master mix (Applied Biosystems; 4385612) on a QuantStudio3 (Applied Biosystems) real-time PCR system. The results were analyzed using the ΔΔCT method with HPRT1 as a reference.

### Myosin heavy chain immunostaining and quantification

Immunostaining and quantification of MHC positive myotubes was performed as previously described [1], and in the Supplemental Methods. Briefly, myoblast cultures were fixed in ice-cold methanol and immunostained for MHC (Developmental Studies Hybridoma Bank MF-20; 1:30) with DAPI counterstain. Two 20x images were obtained from each of two technical replicates for a total of four images per condition, which were analyzed and averaged. MHC area fraction was calculated as the percentage of MHC+ pixels relative to total image pixels and fusion index was calculated as the number of nuclei in MHC+ structures relative to total nuclei in the image.

### Statistical analysis

Study participant characteristics were compared between ND and DIA groups by *t*-test. For experimental data, with the exception of RNAseq data analysis (described above), all between-group comparisons were made with 2- or 3-way ANOVA including within-subjects matching (IMAT vs SQ); comparisons between individual groups were assessed with Fisher’s LSD. Simple linear regression of individual normalized gene counts against hemoglobin A1c (HbA1c) was performed in MATLAB and pathway analysis was performed on genes with significant linear relationships using KEGG mapper. All data were analyzed in GraphPad Prism. Specific statistical tests and numbers per group are reported in each figure or table legend. All data is plotted as mean ± SD.

## Results

### IMAT is morphologically and transcriptionally different from SQ

**Fig. 1:**
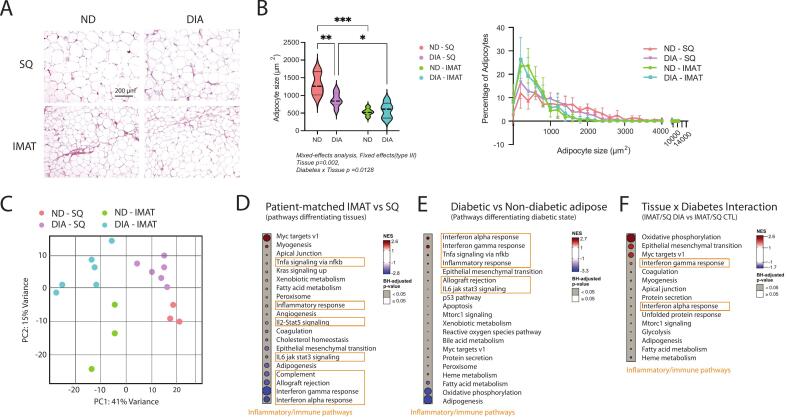
**IMAT is morphologically and transcriptionally different from SQ. (A)** Representative images of SQ and IMAT, stained with H&E from diabetic (DIA) and non-diabetic (ND) participants. Arrows denote small diameter muscle fibers within IMAT samples. **(B)** Quantification of adipocyte average size (top) and size distribution (bottom) across groups. N = 10 **(C)** Principal component analysis (PCA) of RNAseq data; each point represents an individual patient. **(D-F)** Gene set enrichment analysis (GSEA) contrasting **(D)** all IMAT vs SQ, **(E)** all diabetic vs non-diabetic, and **(F)** tissue × diabetic interactions. Inflammatory/immune pathways are highlighted in orange boxes. Statistical analyses (B) Mixed-effect ANOVA *** p < 0.005, ** p < 0.01, *p < 0.05. (For interpretation of the references to colour in this figure legend, the reader is referred to the web version of this article.)

We collected biopsies of IMAT and SQ fat from 13 individuals undergoing elective below-knee amputation surgery, 7 with clinically diagnosed type 2 diabetes (DIA) and 6 without diabetes (ND). There were no significant differences between groups in sex distribution, age or BMI (Table 1). Participants in the DIA group had significantly higher fasting glucose and hemoglobin A1c and represented a range of glycemic control (HbA1c [5.5–8]) due to variations in medication management. Cross-sections of fixed SQ and IMAT from both groups contained primarily unilocular adipocytes typical of white fat; however, IMAT adipocytes were notably smaller than SQ in both DIA and ND samples (Fig. 1A-B). Additionally, SQ adipocytes were significantly smaller in DIA compared with ND samples, while IMAT adipocyte size was not different by diabetes state (Fig. 1B), suggesting that IMAT is uniquely insensitive to diabetes-related pressures on adipocyte size. On several sections, the trace presence of muscle fibers was noted (Supplemental Fig. 1). This microscopic muscle contamination especially in obese or diabetic individuals is also noted in the literature, and considered largely unavoidable [16]. To enhance our ability to identify adipose-centric DEGs and pathways in our RNA sequencing analysis, muscle-specific genes identified by the Human Protein Atlas were excluded from raw RNA counts prior to differential expression analyses.

**Table 1:** Study participant characteristics. statistical analyses: Student’s *t*-test. * p < 0.05 ND vs DIA.

**Parameter**		**ND**	**DIA**	**p-value**
**N (M/F)**		6 (3/3)	7 (4/3)	
**Age (y)**		45.5 ± 11.2	55.6 ± 9.9	0.113
**BMI (kg/m^2^)**		29.7 ± 2.1	31.2 ± 6.6	0.600
**Fasting Glucose (mg/dL)†**		102.3 ± 21.1	139.6 ± 28.4 *	0.015
**HbA1c†**		5.3 ± 0.3	6.5 ± 0.9 *	0.026
	
**Diabetes Medications**		**NA**	Metformin (2), SGLT2 inhibit (3), Insulin (2)	
**Comorbidities**		HTN (3) CKD (0) COPD (1) PN (0)	HTN (4) CKD (2) COPD (1) PN (5)	
**Reason for Amputation**		CP (4) SF (2) DU (0) CH (0)	CP (2) SF (0) DU (1) CH (4)	

† only available in 4 participants from the ND group.

(HTN) hypertension, (CKD) chronic kidney disease, (COPD) chronic obstructive pulmonary disease, (PN) peripheral neuropathy, (CP) chronic pain due to past orthpaedic injury, (SF) surgical failure to restore weightbearing structure, (DU) diabetic ulcer, (CH) charcot deformity.

Principal Component Analysis (PCA) of muscle-subtracted RNAseq highlighted distinct transcriptional profiles between IMAT and SQ, which separated on PC1 (Fig. 1C). Hallmark Gene set enrichment analyses (GSEA) of patient-matched SQ vs IMAT identified these transcriptional differences to be defined largely by enrichment of inflammatory/immune pathways in SQ compared with IMAT (Fig. 1D; orange boxes). DIA samples were transcriptionally distinct from ND as well, separating along PC2 (Fig. 1C) and were largely characterized by enrichment of inflammatory/immune pathways in DIA (Fig. 1E; orange boxes). Pathways featuring a tissue by diabetes interaction were a mix of inflammatory/immune, metabolic and fate regulatory pathways (Fig. 1F). Together, this suggests IMAT is a unique adipose depot with some conserved and some unique responses to diabetes.

### SQ response to poor glycemic control features inflammatory enrichment while IMAT response features metabolic adaptation

**Fig. 2:**
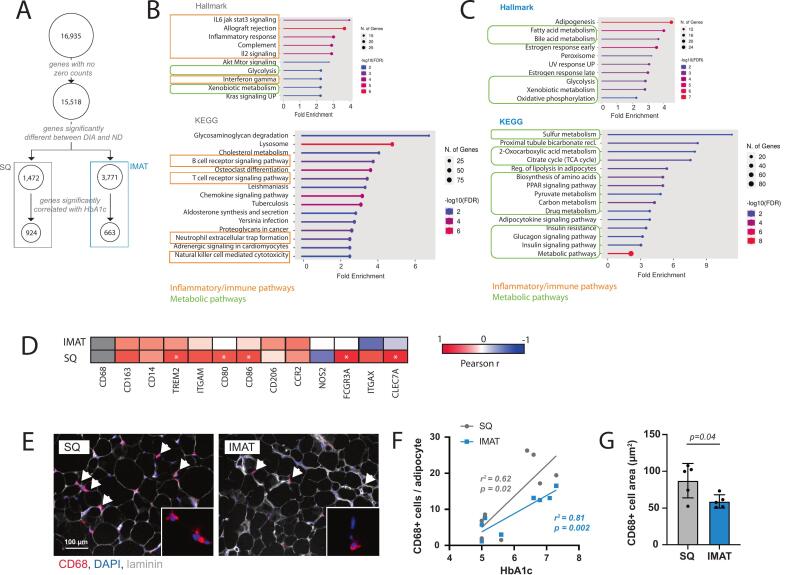
**IMAT and SQ respond differentially to diabetes. (A)** Schematic workflow to filter for diabetic (DIA) vs non-diabetic (ND) differentially expressed genes (DEGs) correlated with HbA1c. **(B-C)** Hallmark and KEGG pathway analysis of HbA1c correlated DIA vs ND DEGs in SQ (B) and IMAT (C). Orange boxes denote inflammatory/immune pathways and green rounded boxes denote metabolic pathways. **(D)** Pearson correlation matrix for macrophages marker gene expression against glycemic control (HbA1c) in IMAT and SQ samples. Asterisks indicate p < 0.05 for the correlation, while boxes are colored by Pearson’s r value. **(E)** Representative images of macrophage identification (arrows) by CD68 immunostaining (red) in SQ and IMAT adipose sections. **(F)** Simple linear regression of CD68+ cells per adipocyte against participant HbA1c for SQ (gray) and IMAT (blue) sections. **(G)** CD68+ cell areas measured in SQ and IMAT sections from participants in the DIA group. Statistical analyses (F) Simple linear regression, (G) Unpaired *t*-test. (For interpretation of the references to colour in this figure legend, the reader is referred to the web version of this article.)

Because DIA participants exhibited a range of glycemic control, we conducted additional analyses on SQ and IMAT DEGs (DIA vs ND) to more deeply probe how IMAT and SQ transcriptional profiles are impacted by this factor. Of the DEGs significantly different between ND and DIA groups, 63 % and 18 % had normalized counts significantly correlated with Hemoglobin A1c (HbA1c) in SQ and IMAT respectively (Fig. 2A). Hallmark and KEGG pathway analyses indicated these genes primarily comprised inflammatory pathways in SQ (Fig. 2B; orange boxes) and metabolic pathways in IMAT (Fig. 2C; green boxes). Notably, these results align with literature reports of an inflammatory transcriptional signature in DIA SQ [17,18] and with the sole report of transcriptional changes in DIA IMAT, with 6 out of our top 15 IMAT KEGG pathways conserved between studies [19]. As the presence of inflammatory macrophages is linked to the inflammatory transcriptional profile in SQ fat [17,18], we investigated the relationship between markers of immune cells and macrophages altered in metabolic disorders and obesity [20,21] and HbA1c across participants. A significant positive correlation was found between HbA1c and TREM2, CD80, CD86, FCGR3A (CD16) and CLEC7A (dectin) in SQ but not in IMAT (Fig. 2D). Immunostaining for CD68 (Fig. 2E), a pan-macrophage marker, found that the density of CD68+ macrophages increased significantly in both SQ and IMAT samples with increasing HbA1c, with a steeper rise in SQ samples (Fig. 2F). Furthermore, CD68+ macrophages were larger in SQ samples than in IMAT samples from the DIA group (Fig. 2G), further supporting phenotypic differences [22]. Together this suggests inflammatory cells and their cytokines may have less influence in IMAT, with adipokines potentially playing a regulatory role (Fig. 2B-C).

### Transcriptional differences between IMAT and SQ include genes encoding secreted adipokines with potential impact on myogenesis

**Fig. 3:**
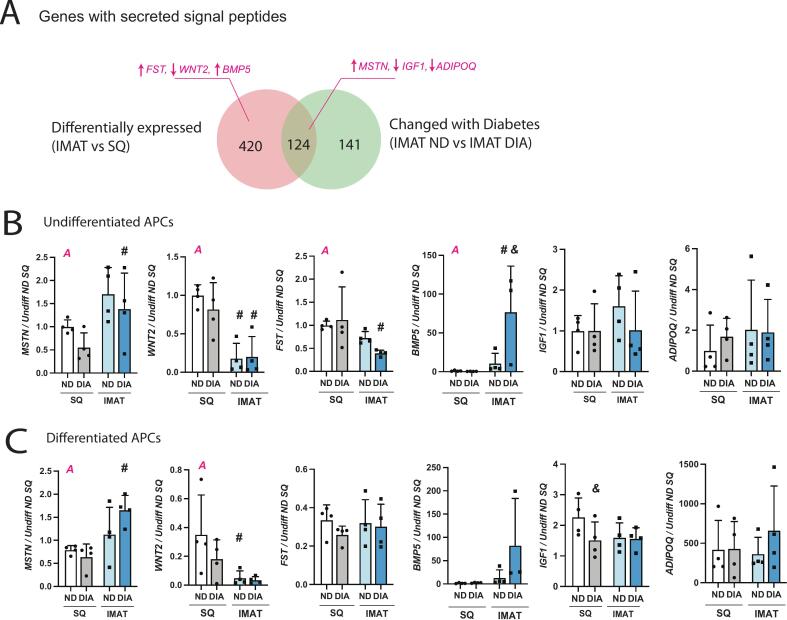
**Transcriptional differences between IMAT and SQ include secreted adipokines with potential impact on myogenesis. (A)** Venn diagram illustrating differentially expressed genes (DEGs) by adipose source encoding secreted proteins that are also DEGs by diabetic state (diabetic (DIA) vs non-diabetic (ND)) and/or correlate with HbA1c. Selected secretory genes with known regulatory action on myogenesis are called out in pink with arrows denoting whether they are up- or down-regulated in IMAT. **(B-C)** qPCR of selected secretory genes in undifferentiated adipose progenitor cells (APCs) (B) and adipogenically differentiated APCs (C). All expression values are normalized to HPRT1 and then to undifferentiated (Undiff) ND SQ to illustrate how expression of each gene changes upon differentiation. Statistical analyses: (B-C) 2-way ANOVA; A indicates main effect of adipose source (IMAT vs SQ), D indicates main effect of diabetes status (ND vs DIA) and I indicates an adipose source x diabetes status interaction. Post-test comparison by groups: # indicates significantly different from SQ with the same diabetes status, & indicates significantly different from ND, same adipose source. #,& p<0.05. (For interpretation of the references to colour in this figure legend, the reader is referred to the web version of this article.)

The genes that were differentially expressed between adipose types (IMAT vs SQ) included 544 genes with secreted signal peptides, which is a tag identifying genes expected to encode secreted proteins (Fig. 3A). Of these, 124 (23 %) are also different between IMAT groups − IMAT DIA vs. ND samples. Some of these genes encode proteins that potentially influence myogenesis (Supplemental Table 1). We then sought to explore whether these differences were retained in isolated adipose progenitor cells (APCs), both to comment on the cellular origin of the tissue-level differences and to determine whether their impact on myogenesis could be examined in a culture model. We selected 6 representative candidates (Fig. 3A; pink arrows denote up or down regulation in IMAT vs SQ). In some of the candidates, patterns from RNAseq were retained in undifferentiated and/or adipogenically differentiated APCs, while others were not (Fig. 3, Fig. 3). Expression of myostatin (MSTN), an inhibitor of myogenesis [23], was elevated in IMAT compared with SQ in both bulk RNAseq and APC qPCR. Expression of Wnt family member 2 (WNT2), a member of the Wnt/β-catenin pathway that could promote myogenesis [24], was reduced in IMAT compared with SQ in both datasets. However, some observations were not consistent with RNAseq data. For example, follistatin (FST), which counteracts myostatin, was elevated in IMAT bulk tissue by RNAseq but found to be reduced in undifferentiated IMAT APCs. Bone Morphogenetic Protein 5 (BMP5) was increased in IMAT in bulk RNAseq data but only in DIA IMAT APCs. Additionally, Insulin-like Growth Factor 1 (IGF-1) and Adiponectin (ADIPOQ) showed no significant differences between APC groups. Candidates with matching results between intact tissue and isolated APCs suggests that APCs contribute to their secretion from adipose tissue and effects on other cell types could be explored through co-culture studies.

### Differentiating human myoblasts are agnostic to the source of APC conditioned media

**Fig. 4:**
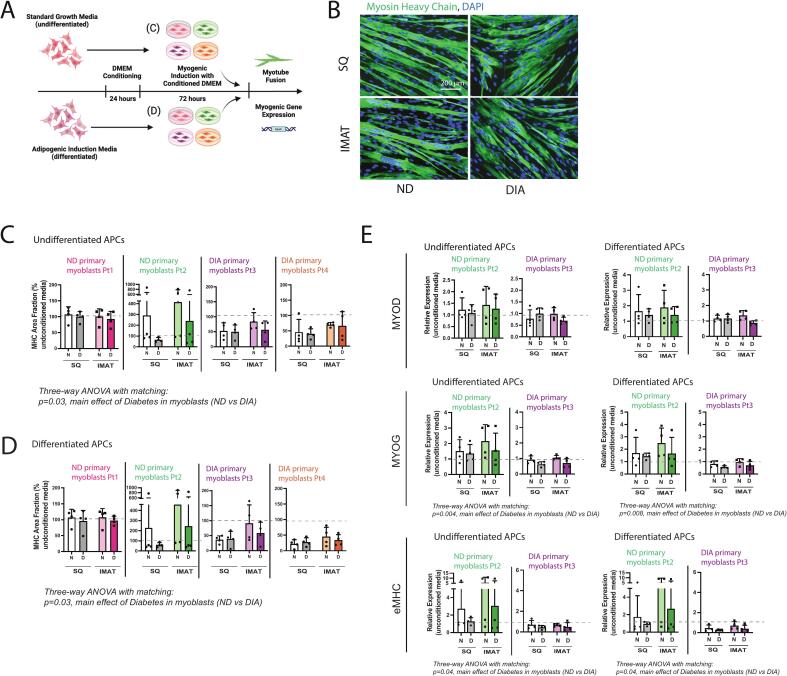
**APC-conditioned media reduces fusion of DIA myoblasts across all APC sources. (A)** Experimental design for assessing myogenesis in conditioned media. Adipose progenitor cells (APCs) from 9 participants were either maintained undifferentiated or adipogenically differentiated and then used to condition DMEM which was then applied with myogenic induction components to separate cultures of myoblasts from 4 participants. Participants 1 and 2 (Pt1 and Pt2) were in the non-diabetic (ND) group and Participants 3 and 4 (Pt3 and Pt4) were in the diabetic (DIA) group. **(B)** Representative myosin heavy chain (MHC) immunostaining (green) of myotube fusion as the final readout of myogenesis. **(C-D)** Quantification of MHC area fraction of Pt1-4 myoblasts treated with conditioned media from undifferentiated (C) and differentiated (D) APCs from SQ (gray/white) and IMAT (colors) from non-diabetic (N) and diabetic (D) participants. **(E)** qPCR of myogenesis markers, MyoD, MyoG, and eMHC in conditioned media treated myoblasts (E). Statistical analyses: (C-E) 3 way ANOVA (with Pt1/Pt2 and Pt3/Pt4 data combined in C-D) to assess the effect of APC source (SQ vs. IMAT), APC diabetes status (ND vs. DIA) and myoblast diabetes status (ND vs. DIA). (For interpretation of the references to colour in this figure legend, the reader is referred to the web version of this article.)

To explore the effect of APC secreted signals on myogenesis, we conditioned DMEM with SQ and IMAT-isolated APCs from 9 participants (4 ND and 5 DIA), generated myogenic induction media with the conditioned DMEM and applied it to myoblasts isolated from 4 participants (2 ND and 2 DIA) (Fig. 4A). Myogenic capacity was assessed by measuring MHC expression in myotubes after treatment with undifferentiated/differentiated APC-conditioned myogenic induction media (Fig. 4B). Notably, the myogenic capacity of myoblasts from each individual and their response to APCs conditioned media varied dramatically across participants (Supplemental Fig. 2A) and thus myogenic capacity of each individual’s myoblasts is normalized to their control condition in unconditioned media. Surprisingly, we observed no main effects of APC source or APC diabetes condition on myogenesis in media conditioned by either undifferentiated or differentiated APCs (Fig. 4, Fig. 4). However, DIA myoblasts exhibited a modest reduction in myogenesis with APC-conditioned media, regardless of APC source, suggesting that DIA myoblasts may be more sensitive to adipose secreted factors and thus, that the IMAT secretome may be more detrimental in diabetes without necessarily changing in composition. This same effect was observed for the fusion index (fraction of nuclei in myotubes) (Supplemental Fig. 2B). Expression of myogenic genes, myogenin (MYOG) and embryonic myosin heavy chain (eMHC), in the treated myoblasts from participant 2 (ND) and 3 (DIA) aligned with the immunostaining quantification (Fig. 4E).

### IMAT does not exhibit features of Brown/Beige adipose tissue

**Fig. 5:**
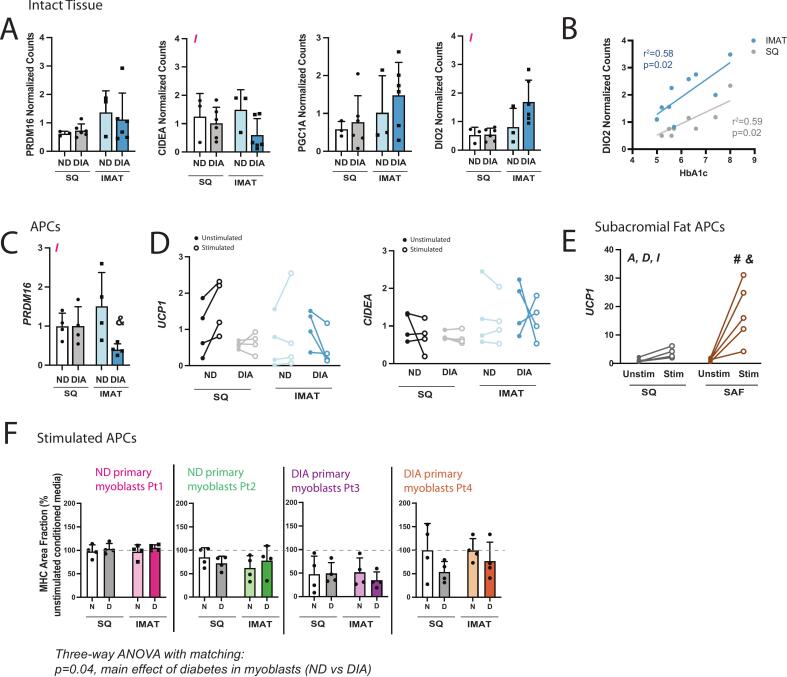
**IMAT does not exhibit brown/beige features. (A)** Normalized counts, determined by the analysis package DESeq2, of selected markers for human BAT. **(B)** Correlation of DIO2 normalized counts with HbA1c in IMAT and SQ. **(C)** Expression of PRDM16 in undifferentiated SQ and IMAT adipose progenitor cells (APCs) by qPCR. **(D)** Change in UCP1 and CIDEA expression in differentiated APCs in response to browning stimulation by isoproterenol by qPCR. **(E)** UCP-1 expression in subacromial fat (SAF) isolated APCs in response to browning stimulation by isoproterenol, validating browning stimulation. **(F)** Quantification of MHC area fraction in myoblast treated with stimulated/non-stimulated APC conditioned media from non-diabetic ND (N) and diabetic DIA (D) participants. Statistical analyses: (A) two-way ANOVA, A indicates main effect of adipose source (IMAT vs SQ), D indicates main effect of diabetes status (ND vs DIA) and I indicates an adipose source x diabetes status interaction, (B) Simple linear regression, (C) two-way ANOVA, (D) three-way ANOVA to assess the effect of of APC source (SQ v IMAT), APC diabetes status (ND v DIA) and stimulation (closed v open symbols), (E) two-way ANOVA with Fisher’s LSD comparison between groups; # p < 0.05 compared with SQ Stim, & p < 0.05 compared with SAF Unstim. (F) three-way ANOVA with Pt1/Pt2 and Pt3/Pt4 data combined to assess the effect of APC source (SQ vs. IMAT), APC diabetes status (ND vs. DIA) and myoblast diabetes status (ND vs. DIA).

As smaller adipocyte size and altered secretome are associated with beige adipose tissue in humans [25], and the browning capacity of IMAT progenitor cells has been suggested [26], we sought to investigate markers of brown/beige adipose tissue in the intact tissue and in stimulated progenitors. In RNAseq data from intact IMAT, UCP1 expression was not detectable. Other selected markers (PRDM16, CIDEA, PGC1A, DIO2) for BAT in humans did not show significant differences between IMAT and SQ. However, CIDEA and DIO2 displayed a significant adipose source by diabetes interaction, suggesting they may be differentially altered by diabetes (Fig. 5A). Interestingly, DIO2 was significantly correlated with HbA1c in both IMAT and SQ (Fig. 5B), further supporting that differences are driven by diabetes. In isolated APCs, the expression of PRDM16, a marker for brown/beige adipose progenitors, also exhibited a significant adipose source by diabetes interaction, with IMAT DIA APCs having significantly lower expression than IMAT ND APCs (Fig. 5C). When differentiated APCs were stimulated with a browning reagent (isoproterenol), the expression of UCP-1 and CIDEA (transcriptional markers of the browning response) was not changed (Fig. 5D). By contrast, a robust increase in UCP-1 expression was observed in subacromial fat (SAF) isolated APCs, a validated human beige adipose depot with in-vitro browning capacity [15] (Fig. 5E). We also collected DMEM conditioned by stimulated APCs to determine whether stimulation would impact regulation of myogenesis. There was no improvement in myogenesis in myoblasts from any participant when treated with stimulated APC conditioned media. In fact, stimulated conditioned media further decreased fusion in diabetic myoblasts specifically (Fig. 5F). Overall, IMAT does not possess transcriptional features of brown/beige adipose tissue either in vitro or in intact tissue and stimulation with a browning agent does not improve myogenic cross-talk between IMAT and myoblasts.

## Discussion

The potential impact of IMAT on the progression of muscle pathology has been a topic of discussion for decades. The close juxtaposition of IMAT adipocytes and myofibers combined with our advancing understanding of adipose signaling, has spawned hypotheses that IMAT directly impacts myofiber function through paracrine signaling [10,27]. Unbiased transcriptomic and secretomic data combined with co-culture experiments support this to be the case for the development of myofiber insulin resistance in diabetes [11,19,28], however, myofiber insulin resistance is only one piece of a complex diabetic muscle pathology which also features fibrosis, necrosis and impaired regenerative capacity, especially in chronic diabetes with co-morbidities [1]. Here, we have taken a similar approach to provide insight into the role of IMAT paracrine signaling in muscle regeneration in highly pathologic muscle of diabetes. We found that IMAT is morphologically and transcriptionally unique compared with SQ adipose and identified transcriptional differences in the DIA group. Interestingly, we found that IMAT was characterized by a lower inflammatory transcriptional profile than SQ across all participants and while DIA SQ exhibited signs of increased inflammatory cell infiltration with worsening glycemic control, IMAT did not. This suggests that in these individuals, IMAT paracrine signaling may be mediated more by adipokines than inflammatory cytokines. While we identified candidate adipokines with potential pro- and anti-myogenic action that are unique to IMAT and/or altered with diabetes, ultimately we found that media conditioned by differentiated IMAT progenitors did not uniquely impact myoblast fusion. Surprisingly, we instead found that the diabetic status of the myoblast donors most influenced fusion rates, where DIA myoblasts exhibited reduced fusion in response to all adipose progenitor (APC) conditioned media. Thus, we conclude that IMAT has the potential to decrease myoblast differentiation via paracrine signaling but that this effect is not modeled by APCs in culture, suggesting contribution by other IMAT cells. However, increased sensitivity of DIA myoblasts to APC conditioned media suggests that IMAT could impair differentiation even in the absence of secretome changes.

To our knowledge, only one other dataset has been generated to transcriptionally profile human IMAT [19,29]. In line with that study, we find that IMAT has a unique transcriptional profile compared with SQ, which shifts in diabetes (Fig. 1C). Furthermore, several of the pathways identified in that dataset as correlated with insulin sensitivity in IMAT [19] are also enriched in our geneset of features significantly correlated with glycemic control (HbA1c; Fig. 2C). These include metabolic pathways such as oxidative phosphorylation and insulin signaling as well as adipocytokine signaling. However, in contrast to their dataset, we do not find enrichment in pathways related to inflammation in IMAT (e.g. JAK/STAT, B- and T-cell signaling pathways) and instead find this uniquely in SQ. This could be due to differences in the range of participant systemic health as that dataset includes IMAT from athletes and lean individuals as well as obese and diabetic individuals, where ours comprises individuals with similar (overweight or obese) BMI and limited mobility with and without clinically diagnosed diabetes, and these individuals may have elevated IMAT inflammation across the board relative to true healthy controls. Indeed, while Sachs et al. finds significant changes in inflammatory macrophage markers (e.g. CD11c, CD68, dectin) over all participants, these are minimally different between the obese and diabetic groups, which aligns with our findings (Fig. 2, Fig. 2). Notably, however, we do find elevated inflammatory pathways in SQ adipose of those with diagnosed diabetes and more CD68+ macrophages, which suggests that there are differences in inflammation in other adipose depots between these groups.

Additionally, our within-subject study design enabled us to compare IMAT and SQ in the same individual, finding that the SQ transcriptome had enriched inflammatory pathways compared with IMAT. This is in contrast to a recent study which finds higher secretion of inflammatory cytokines from explants of IMAT compared with SQ fat [11]. Our finding of smaller adipocyte size in IMAT compared with SQ (Fig. 1B) may offer insight into this discrepancy since this suggests that IMAT has a higher density of cells per unit mass and thus may secrete higher levels of cytokines per unit mass, but not per cell. In addition to elevated secretion of pro-inflammatory cytokines by IMAT compared with SQ and visceral fat, this study also found significantly elevated secretion of anti-inflammatory cytokines, adipokines and eicosanoids, with nothing significantly reduced, suggesting that secretion of cytokines may be higher in explanted IMAT across the board. If true, this suggests that signaling from a given quantity of IMAT could have more influence on muscle than a similar quantity of SQ or visceral fat.

The dearth of description of IMAT at the cellular level (morphology, transcriptome, secretome) has arisen in large part from the difficulty in isolating it in sufficient quantities for analysis. In fact, there is more cellular level analysis of IMAT in livestock where it can be dissected from whole muscles. This literature similarly finds smaller IMAT adipocytes [30,31] and a unique transcriptional profile [30,32] compared with SQ. In livestock with obesity, evidence for inflammation in IMAT is conflicting. Studies in obese pigs found methylation patterns on key inflammatory genes to be similar between IMAT and visceral fat [33] with higher expression of those genes in IMAT and visceral fat compared with SQ [34]. However, studies in obese cattle have found higher macrophage infiltration in visceral fat only with no difference between IMAT and SQ [35] and a downregulation of immune related transcriptional pathways in IMAT compared with visceral and SQ [36,37]. In this study, we have circumvented the issue of limited IMAT from muscle biopsy by acquiring large muscle volumes from individuals undergoing below-knee amputation, which has enabled transcriptional profiling, histological analysis and progenitor cell isolation from the same sample. It also enabled us to identify and preferentially dissect intrafascicular rather than the more abundant perifascicular IMAT. The differences between these anatomical IMAT regions have largely not been explored, but intrafascicular IMAT is thought to reflect a state of muscle degeneration, rather than simple adipose expansion [38] and this may offer an additional explanation for the discrepancy between studies.

Generally, the myogenesis-related cytokine expression in IMAT is suggestive that IMAT would impair myogenesis (e.g. increased myostatin, decreased IGF-1, decreased adiponectin; Fig. 3A and Supplemental Table 1). These changes are associated with obesity in SQ adipose [[39], [40], [41]] and conditioned media from obese SQ adipose can reduce myogenesis in cultured myoblasts [9,12,13]. However, despite differential expression of these cytokines in IMAT vs SQ, IMAT APC conditioned media did not uniquely affect myoblast fusion (Fig. 4). This could be because some differential adipokine expression (e.g. IGF-1, adiponectin) was not retained in cultured APCs, either because APCs were not the source of those differences in the intact tissues, they require an intact in-vivo environment to express them or because time spent in culture erased them. Conditioning media with intact IMAT, which contains adipocytes, APCs and other cell types [42], may be required to fully assess the unique effect of its secreted cytokines, but offers less potential for sustained mechanistic exploration. Importantly, we do find an effect of APC conditioned media on diabetic myoblasts, offering an avenue for future mechanistic insight into IMAT-myoblast cross-talk, specific to diabetes (Fig. 4). Our observation that APC conditioned media decreased diabetic myoblast fusion, but not non-diabetic myoblast fusion was unexpected. It suggests an intrinsic sensitivity in diabetic myoblasts that could make them uniquely dysfunctional in a muscle environment with high IMAT. To our knowledge, only our previous study in this same cohort [1] has compared in vitro fusion between diabetic and non-diabetic human myoblasts, which found no difference in unconditioned media. However, limited data supports the conceptual framework that conditions of the donor myoblasts (e.g. age) may impact their sensitivity to adipokines [9]. Together this highlights the importance of using primary cells from multiple individuals, across conditions to fully elucidate pathological signaling. However, it should also be noted that muscle regeneration is the result of coordinated action of many cell types including immune cells, fibro-adipogenic progenitors (FAPs) and other supporting cells (e.g. endothelial cells, pericytes) and thus in vitro myoblast fusion reflects only one piece of the puzzle. While the in vivo regeneration assays used to demonstrate impaired regeneration in diabetic mouse models [[43], [44], [45]] cannot be used in people, advances in muscle-on-a-chip models may soon enable the study of more complex regeneration in vitro.

It is important to note that, as IMAT APCs are adipogenic progenitors derived from muscle, they are comparable to FAPs. Undifferentiated murine FAPs play a critical role in muscle regeneration [46,47] and promote myogenesis in cultured myoblasts [47]. We did not observe this effect in our conditioned media experiments where undifferentiated IMAT APC conditioned media either had no effect or a negative effect on myogenesis. This could reflect species specificity or, more likely, altered signaling in APCs isolated from high-IMAT areas. Some evidence in mice also suggests that IMAT is a beige adipose depot capable of browning in response to stimuli [48,49], while other evidence suggests limited browning potential for IMAT with higher potential in epimuscular adipose tissue [50]. Our data suggests that this latter difference may extend to humans since we do not find elevated expression of brown/beige fat related genes in IMAT, or any response of cultured APCs to a browning stimulation (Fig. 5A-D), which stands in contrast to subacromial fat which is an epi-muscular adipose in the shoulder [15] (Fig. 5E). A lack of browning capacity in our IMAT APCs agrees with literature findings that IMAT from obese adults most closely resembles white adipose [26,28].

The purpose of this study is to understand the features of IMAT in clinical populations with major orthopaedic complications of the lower extremity and thus caution should be employed in extrapolating these results to other populations including those with well-managed diabetes and healthy controls. The range in glycemic control (HbA1c) in our DIA group reflects differences in clinical management of diabetes at the time of amputation as individuals were not excluded from this study based on medications. While an oral glucose tolerance test or insulin tolerance test would provide additional insight into insulin resistance, this study did not have access to participants prior to surgery and thus relies on the clinical diagnosis of diabetes and stratifies individuals based on glycemic control recorded in the participant’s medical record. This is both a limitation and strength of this study. While it is not a traditional prospectively-enrolled study with tight inclusion/exclusion criteria, it reflects the range of diabetes management typically encountered in the orthopaedic or rehabilitative setting and utilizing a within-subjects design and considering glycemic control as a moderator, these data have shed light on how IMAT changes across this range.

Several additional limitations should be considered when interpreting this data. First, IMAT was only compared with SQ, when evidence suggests it may be more like visceral adipose [27]. We were unable to acquire visceral adipose from these participants and opted for the increased power of a within-subjects design. Future studies comparing human IMAT to SQ, visceral and epi-muscular depots will further characterize the unique features of IMAT. Second, adipose samples were taken from an intrinsic muscle of the foot which may not be representative of other musculature. This muscle was chosen because of its relatively high levels of IMAT in our control group (compared with the gastrocnemius) and due to its proposed role in contributing to foot deformity and disability in people with type 2 diabetes [51]. Third, the DIA group had a higher incidence of co-morbidities, peripheral neuropathy in particular. Peripheral neuropathy has a well-defined effect on myofiber structure and function, but its effect on IMAT cellular composition and signaling is virtually unexplored. At this time, we can only speculate on the generalizability of these findings to individuals with well controlled diabetes or other muscle pathologies. Finally, the bulk RNA sequencing approach utilized here cannot identify the cellular source of the transcriptional changes, which could arise from adipocytes, APCs, immune or endothelial cells among others. Future studies using single-cell sequencing techniques will be valuable in dissecting the individual cellular contributors between adipose types.

## Conclusion

In conclusion, this study advances our understanding of the unique features of IMAT, how it changes with advanced diabetic muscle pathology and how it may impact muscle regeneration. We find that IMAT is a morphologically and transcriptionally unique adipose compared with SQ, characterized by lower levels of inflammation in advanced diabetic muscle pathology. IMAT expresses elevated levels of several secreted signals that could impair regeneration, but conditioned media from cultured progenitor cells only impaired myogenesis in DIA myoblasts, suggesting that anti-myogenic cross-talk between IMAT and myoblasts may be mediated by an increased sensitivity of diabetic myoblasts to adipokines rather than a shift in the secretory profile of IMAT progenitors. Future studies are needed to identify key cytokines mediating this effect and identify their cellular source within IMAT.

## CRediT authorship contribution statement

**Chang Gui:** Writing – review & editing, Writing – original draft, Methodology, Investigation, Formal analysis, Data curation, Conceptualization. **Dakota R. Kamm:** Writing – review & editing, Methodology, Investigation, Formal analysis. **Jeremie L.A. Ferey:** Writing – review & editing, Methodology, Investigation, Formal analysis. **Kathryn L. Bohnert:** Writing – review & editing, Methodology, Investigation, Data curation. **Jeremy J. McCormick:** Writing – review & editing, Resources, Methodology, Conceptualization. **Mary K. Hastings:** Writing – review & editing, Project administration, Methodology, Conceptualization. **Gretchen A. Meyer:** Writing – review & editing, Writing – original draft, Visualization, Validation, Supervision, Software, Resources, Project administration, Methodology, Investigation, Funding acquisition, Formal analysis, Data curation, Conceptualization.

## Declaration of competing interest

The authors declare that they have no known competing financial interests or personal relationships that could have appeared to influence the work reported in this paper.
