# Supplementary material for: Insights into intramuscular adipose–muscle signaling in the diabetic lower extremity
Source: J Clin Transl Endocrinol. 2025 Oct 28;42:100422. doi: 10.1016/j.jcte.2025.100422 (PMC12617770; doi:10.1016/j.jcte.2025.100422)
Supplement: Supplementary Data 1 [file mmc1.docx]

# Supplementary Methods

*RNA Isolation and Sequencing*

Total RNA integrity was determined using Agilent Bioanalyzer or 4200 Tapestation. Library preparation was performed with 500ng to 1ug of total RNA. Ribosomal RNA was removed by an RNase-H method using RiboErase kits (Kapa Biosystems). mRNA was then fragmented in reverse transcriptase buffer and heating to 94 degrees for 8 minutes. mRNA was reverse transcribed to yield cDNA using SuperScript III RT enzyme (Life Technologies, per manufacturer's instructions) and random hexamers. A second strand reaction was performed to yield ds-cDNA. cDNA was blunt ended, had an A base added to the 3' ends, and then had Illumina sequencing adapters ligated to the ends. Ligated fragments were then amplified for 12-15 cycles using primers incorporating unique dual index tags. Fragments were sequenced on an Illumina NovaSeq-6000 using paired end reads extending 150 bases.

*Adipose Progenitor Cell Isolation and Culture*

A portion of each adipose sample was digested for adipose progenitor cell (APC) isolation and culture, as previously described (1). Briefly, adipose samples were minced and incubated in a 2% collagenase solution for 30 min followed by further mechanical dissociation by pipetting and filtration. Isolated cells were plated and expanded in standard growth media (10% FBS, 1% Penicillin/Streptomycin in low-glucose DMEM) until passage 2-4 which selects for the APC population (2). APCs were grown to confluence in 6-well plates and assigned to one of three treatment groups: 1) undifferentiated – maintained in standard growth media, 2) differentiated – treated with adipogenic induction media (10% FBS, 1% Penicillin/Streptomycin, 0.5 mM 3-isobutyl-1-methylxanthine (IBMX), 0.1 uM dexamethasone, 1.7 uM insulin, 10 uM indomethacin in low-glucose DMEM) for 6-8 days followed by 6-8 days of adipogenic maintenance media (10% FBS, 1% Penicillin/Streptomycin, 0.1 uM dexamethasone, 1.7 uM insulin in low-glucose DMEM) and 3) differentiated + stimulated – differentiated cultures were treated with 10 uM isoproterenol for 8 hrs prior to DMEM conditioning. The number of days in adipogenic media was varied slightly between participants to match levels of adipogenesis, which was confirmed on 4x and 10x bright field images. Media was changed every 2 days and the total culture time was the same between groups. Following the culture described above, cells were washed three times with PBS and incubated in low-glucose DMEM for 24 hours for conditioning. Conditioned DMEM was used to make myogenic induction media (5% horse serum, 1% Penicillin/Streptomycin in conditioned DMEM) which was applied to myoblast cultures described below. Following DMEM collection, ASC cultures were washed with PBS and collected in Trizol for RNA extraction.

*Myoblast Isolation and Culture*

A portion of each muscle sample was digested for myoblast isolation as previously described (3). Briefly, cells were isolated from human smaples by incubation in digestion solution (0.25% collagenase type I, 0.06 units/mL dispase II in low-glucose DMEM), facilitated with mechanical disruption. Cell suspensions were washed and filtered with DMEM. Filtered cells were plated to isolate adherent cells and then cryobanked. Myoblasts from four participants (2 in the ND group and 2 in the DIA group) were FACS sorted to isolate myoblasts (CD45-/CD31-/CD56+) and myoblasts were expanded to passage 2. Myobalsts were cultured to 70% confluency in a 24-well plate in parallel with APC cultures. Then, myoblasts from each of the four participants were treated with conditioned myogenic induction media (described above) from each of the 9 participants’ APC cultures, resulting in condition-matched (ND APCs : ND myoblasts, DIA APCs : DIA myoblasts) and condition-mismatched (ND APCs : DIA myoblasts, DIA APCs : ND myoblasts) pairings. Myoblasts treated with unconditioned myogenic induction media served as an internal control for each participant. Following three days in the media, with no media change, half of the myoblast cultures were collected in Trizol for RNA extraction while another half were washed with PBS and fixed with pre-chilled methanol for MHC immunostaining.

*RT-qPCR*

The total RNA of APCs and myoblasts was extracted using phenol-chloroform extraction as previously described (1). Briefly, 1/5 volume of chloroform was vigorously mixed with trizoled cell lysate and kept on ice. The mixture was then centrifuged for phase separation and the aqueous phase mixed with equal volume of isopropanol for RNA precipitation and separation by centrifugation. The resulting pellet was washed with 75% ethanol and re-pelleted by centrifugation. Then the pellet was air dried and resuspended in 20 μl DEPC water. RNA quality (260/280>1.8) was confirmed by Nanodrop (Thermo Scientific). cDNA was prepared with MultiScribe reverse transcription kit (Applied Biosystems; 4368814) according to manufacturer’s protocol. Then, qPCR was performed with cDNA and SYBR Green PCR master mix (Applied Biosystems; 4385612). Briefly, 1 μL of cDNA was added into a master mix containing 10 uL Sybr green, 7.7 uL DEPC water, 0.64 uL forward and reverse primer each. All primers listed in Table S1 were designed with the online tool primer-blast. The samples were run in triplicate on a pre-programmed QuantStudio3 (Applied Biosystems) real-time PCR system. The results were analyzed using ΔΔCT methods with HPRT1 as a reference.

**Table S1.** Human qPCR Primer sequences

| Gene | Forward sequence (5’ – 3’) | Reverse sequence (3’ – 5’) |
| --- | --- | --- |
| ADIPOQ | GATGAAGTCCTGTCTTGGAAGG | CAGCACTTAGAGATGGAGTTGG |
| BMP5 | GCAGAAACAGGGGATGGACG | CAACAAGGCTTTGGTACGTGG |
| CIDEA | CATGTATGAGATGTACTCCGTGTC | GAGTAGGACAGGAACCGCAG |
| eMHC | TTGCTGTCTTCTGCTCTCATCC | GGAGCAGCTATGCCGAACAC |
| FST | GGAAAACCTACCGCAATGAA | GAGCTGCCTGGACAGAAAAC |
| IGF1 | ATGCTCTTCAGTTCGTGTGTGG | CAATACATCTCCAGCCTCCTTAGA |
| MSTN | TGGTCATGATCTTGCTGTAACCTT | TGTCTGTTACCTTGACCTCTAAAA |
| MYOD | CGACGGCATGATGGACTACA | TAGTAGGCGCCTTCGTAGCA |
| MYOG | CAGTGCACTGGAGTTCAGCG | TTCATCTGGGAAGGCCACAGA |
| PRDM16 | CACGCAGAACTTCTCACTGC | ATGGGAGCAAATACTGACGG |
| UCP1 | AGGTCCAAGGTGAATGCCC | GCGGTGATTGTTCCCAGGA |
| WNT2 | GCTGGAATTGCAACACCCTG | ACCGCTTTACAGCCTTCCTG |
| HPRT1 | TGCTCGAGATGTGATGAAGG | TCCCCTGTTGACTGGTCATT |

*Myosin Heavy Chain Immunostaining and Quantification*

Immunostaining and quantification of MHC positive myotubes was performed as previously described (3). Briefly, myoblasts were gently washed with PBS and fixed with 0.5mL ice-cold methanol for 10 min. After fixation, myoblasts were incubated with MF-20 (Developmental Studies Hybridoma Bank; 1:30) for 1hr. The plates were gently washed with PBS 3 times. Then, the cells were incubated with 200 ul of Alexafluor 488 goat-anti-mouse secondary antibody in 2% BSA (Invitrogen # A-11001; 1:400) for 20 min. The plate was then washed with PBS twice and the myonuclei were counterstained stained with DAPI. Following staining, one 10x image and two 20x images were taken using the GFP (MHC) and DAPI channels for each well using EVOS Flc microscope (Invitrogen). 2 wells were imaged for each culture condition, for a total of four 20x images which were analyzed and averaged. MHC images were smoothed by Gaussian blur in ImageJ prior to thresholding using Huang’s method in the AutoThreshold package and masking. Masked images were used to determine MHC area fraction as the percentage of positive pixels relative to total pixels. Fusion index was quantified by deleting the inverse mask on the corresponding DAPI image and using Analyze Particles with Watershed to count the number of nuclei within MHC+ myotubes and dividing that by the total number of nuclei in the image.

**
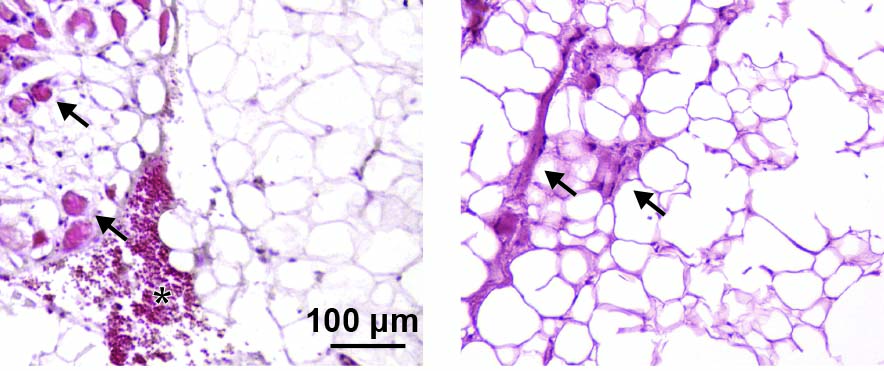
**

**Supplementary Fig. 1. Images of micro myofiber contamination in IMAT samples.** Small caliber myofibers are labeled by arrows in two IMAT samples. Asterisk denotes red blood cell accumulation due to local bleed.


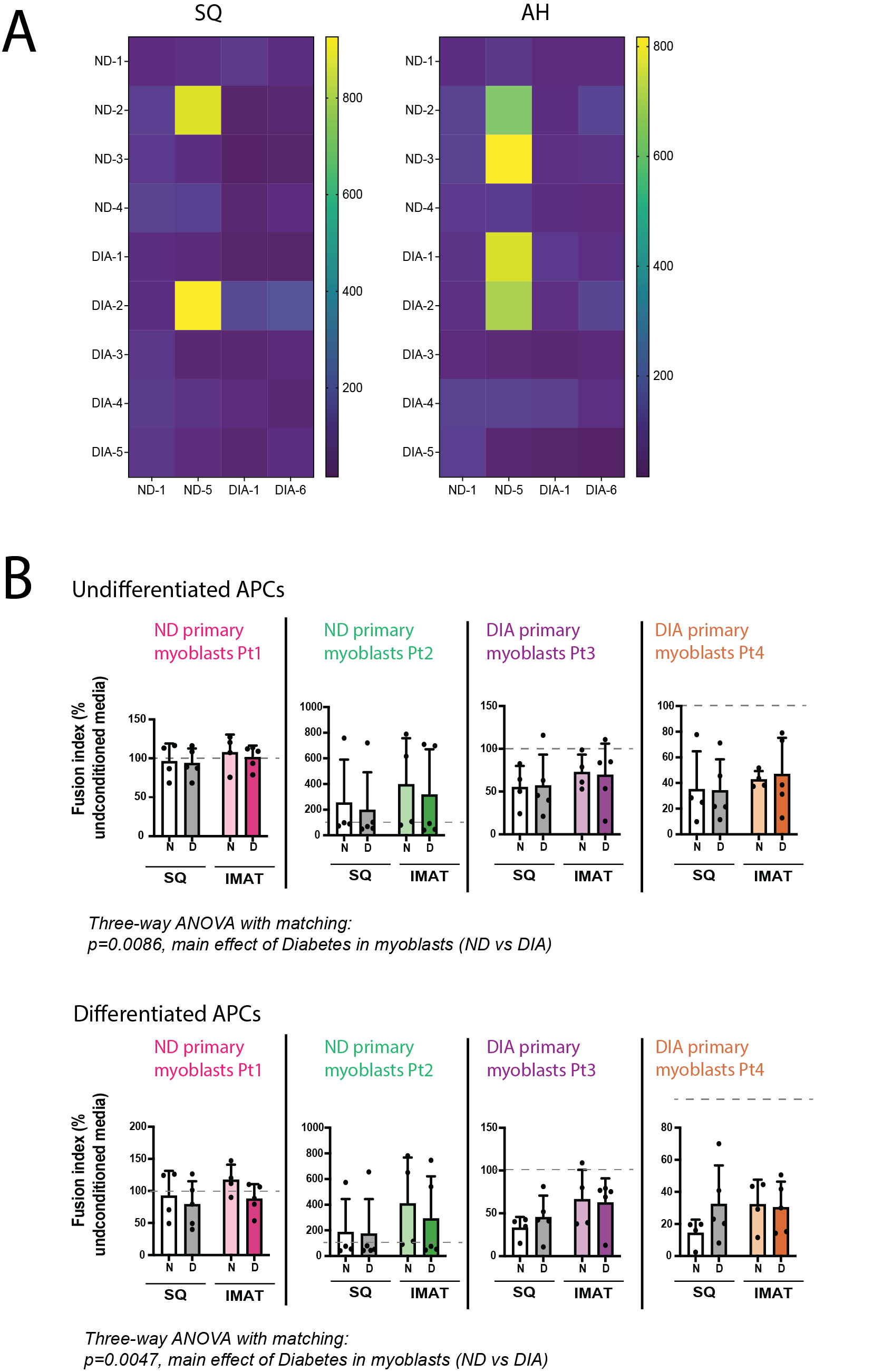


**Supplementary Fig. 2. Additional quantification of APC-myoblast co-culture.** (A) Heatmap of APC conditioned myoblasts MHC area normalized to myoblasts treated with unconditioned myogenic induction media. Each row represents APCs, and each column represents myoblasts. (B) Quantification of Fusion index of Pt1-4 myoblasts treated with conditioned media from undifferentiated (top) and differentiated (bottom) APCs from non-diabetic (N) and diabetic (D) participants.

**Supplemental Table 1.** Genes with secreted signal peptide by comparison. Shading indicates genes that have published action on myogenesis or regeneration

| **IMAT vs SQ p<0.05** | **PMID** | **in IMAT** | | **AH: DIA vs CTL** | **PMID** | **in DIA** |  |  |  |
| --- | --- | --- | --- | --- | --- | --- | --- | --- | --- |
| ADIPOQ | 32003014, 33359123 | down |  | ADIPOQ | 32003014, 33359123 | down |  |  | Predicted to improve myogenesis or regeneration |
| BMP4 | 20689554 | up |  | CCL2 | 36575043 | up |  |  | Mixed or diverging effects |
| IGF1 | 12424223, 11175789 | down |  | IGF1 | 12424223, 11175789 | up |  |  | Predicted to impair myogenesis or regeneration |
| IGFBP3 | 12599210 | down |  | VEGFA | 15509502 | down |  |  |  |
| WNT2 | 25949788 | down |  | FGF8 | 38147545 | up |  |  |  |
| AGRN | 36233091 | down |  | IL15 | 29021612 | up |  |  |  |
| FGF2 | 26074812 | down |  | FGF7 | 38751367 | up |  |  |  |
| GDNF | 32021964 | up |  | CSF3 | 24822049 | up |  |  |  |
| NRG1 | 12788100 | down |  | IGFBP4 | 18258685, 7514606 | up |  |  |  |
| PDGFB | 36521377 | down |  | IL6 | 29778303 | up |  |  |  |
| SFRP1 | 14745964 | up |  | FGF1 | 10595310 | down |  |  |  |
| SFRP4 | 14745964 | up |  | IL17B | 22285818 | up |  |  |  |
| CSF3 | 24822049 | up |  | GDF11 | 26001423 | up |  |  |  |
| TNF | 17151142 | down |  | HGF | 9551084 | up |  |  |  |
| ADAMTS12 | 9417060 | down |  | LEP | 23201486 | down |  |  |  |
| CCN2 | 25261584 | up |  | LIF | 15843032 | up |  |  |  |
| FGF1 | 10595310 | down |  | IL7 | 20089933 | up |  |  |  |
| IL17B | 22285818 | up |  | MSTN | 16079293, 15699335 | up |  |  |  |
| LEP | 23201486 | down |  | ABHD15 |  |  |  |  |  |
| MSTN | 16079293, 15699335 | up |  | AC233755.1 |  |  |  |  |  |
| TNFSF10 | 32645396 | down |  | ACAN |  |  |  |  |  |
| DKK1 | 25949788 | down |  | ADAM23 |  |  |  |  |  |
| TGFB2 | 33531476, 33531466 | up |  | ADAMTS12 |  |  |  |  |  |
| AADACL2 |  |  |  | ADAMTS14 |  |  |  |  |  |
| ABHD15 |  |  |  | ADAMTSL1 |  |  |  |  |  |
| ACE |  |  |  | ADCYAP1 |  |  |  |  |  |
| ACP3 |  |  |  | ADGRE3 |  |  |  |  |  |
| ADA2 |  |  |  | ANKDD1A |  |  |  |  |  |
| ADAM12 |  |  |  | ANXA2 |  |  |  |  |  |
| ADAM23 |  |  |  | AOC3 |  |  |  |  |  |
| ADAM9 |  |  |  | APP |  |  |  |  |  |
| ADAMTS10 |  |  |  | ARTN |  |  |  |  |  |
| ADAMTS13 |  |  |  | BGN |  |  |  |  |  |
| ADAMTS15 |  |  |  | BPIFB4 |  |  |  |  |  |
| ADAMTS17 |  |  |  | BTBD17 |  |  |  |  |  |
| ADAMTS18 |  |  |  | BTD |  |  |  |  |  |
| ADAMTS19 |  |  |  | C1RL |  |  |  |  |  |
| ADAMTS2 |  |  |  | C1S |  |  |  |  |  |
| ADAMTS20 |  |  |  | C1orf54 |  |  |  |  |  |
| ADAMTS4 |  |  |  | C2 |  |  |  |  |  |
| ADAMTS5 |  |  |  | C3 |  |  |  |  |  |
| ADAMTS7 |  |  |  | C4A |  |  |  |  |  |
| ADAMTSL1 |  |  |  | C4B |  |  |  |  |  |
| ADAMTSL3 |  |  |  | C7 |  |  |  |  |  |
| ADAMTSL4 |  |  |  | CCL16 |  |  |  |  |  |
| ADGRB1 |  |  |  | CCL8 |  |  |  |  |  |
| ADGRG1 |  |  |  | CCN4 |  |  |  |  |  |
| ADM |  |  |  | CFB |  |  |  |  |  |
| AEBP1 |  |  |  | CFH |  |  |  |  |  |
| AGER |  |  |  | CFI |  |  |  |  |  |
| AGT |  |  |  | CHSY1 |  |  |  |  |  |
| AIMP1 |  |  |  | CNMD |  |  |  |  |  |
| AKR1B10 |  |  |  | COL10A1 |  |  |  |  |  |
| ALCAM |  |  |  | COL14A1 |  |  |  |  |  |
| AMH |  |  |  | COL18A1 |  |  |  |  |  |
| AMN |  |  |  | COL1A1 |  |  |  |  |  |
| AMY2B |  |  |  | COL1A2 |  |  |  |  |  |
| ANGPT1 |  |  |  | COL21A1 |  |  |  |  |  |
| ANGPTL1 |  |  |  | COL4A6 |  |  |  |  |  |
| ANGPTL2 |  |  |  | COL7A1 |  |  |  |  |  |
| ANGPTL4 |  |  |  | COL8A1 |  |  |  |  |  |
| ANGPTL7 |  |  |  | CPA3 |  |  |  |  |  |
| ANGPTL8 |  |  |  | CPXM2 |  |  |  |  |  |
| ANKDD1A |  |  |  | CRISPLD2 |  |  |  |  |  |
| ANXA1 |  |  |  | CXCL17 |  |  |  |  |  |
| ANXA2 |  |  |  | CXCL5 |  |  |  |  |  |
| AOAH |  |  |  | CYTL1 |  |  |  |  |  |
| AOC3 |  |  |  | DHRSX |  |  |  |  |  |
| APLN |  |  |  | DIPK2A |  |  |  |  |  |
| APOB |  |  |  | DKK2 |  |  |  |  |  |
| APOC1 |  |  |  | DKK3 |  |  |  |  |  |
| APOE |  |  |  | ECM2 |  |  |  |  |  |
| APOL1 |  |  |  | EDIL3 |  |  |  |  |  |
| APOL4 |  |  |  | EMC10 |  |  |  |  |  |
| APOO |  |  |  | EMID1 |  |  |  |  |  |
| ART4 |  |  |  | EMILIN1 |  |  |  |  |  |
| ART5 |  |  |  | EMILIN3 |  |  |  |  |  |
| ASPN |  |  |  | ENPP1 |  |  |  |  |  |
| ATRN |  |  |  | EYS |  |  |  |  |  |
| AVP |  |  |  | FAM20A |  |  |  |  |  |
| AZGP1 |  |  |  | FAS |  |  |  |  |  |
| B2M |  |  |  | FBLN1 |  |  |  |  |  |
| B3GAT1 |  |  |  | FCGR3B |  |  |  |  |  |
| BCHE |  |  |  | FCN1 |  |  |  |  |  |
| BMP1 |  |  |  | FGF2 |  |  |  |  |  |
| BMP5 |  |  |  | FGFR2 |  |  |  |  |  |
| BMPER |  |  |  | FOLR1 |  |  |  |  |  |
| BPI |  |  |  | FSTL3 |  |  |  |  |  |
| BPIFB4 |  |  |  | GAS6 |  |  |  |  |  |
| BTD |  |  |  | GHR |  |  |  |  |  |
| C11orf45 |  |  |  | GREM1 |  |  |  |  |  |
| C15orf61 |  |  |  | HAPLN3 |  |  |  |  |  |
| C16orf89 |  |  |  | HHIPL2 |  |  |  |  |  |
| C17orf67 |  |  |  | HMCN2 |  |  |  |  |  |
| C1QA |  |  |  | HMSD |  |  |  |  |  |
| C1QB |  |  |  | HP |  |  |  |  |  |
| C1QC |  |  |  | IGFBP5 |  |  |  |  |  |
| C1QTNF1 |  |  |  | IGFBP7 |  |  |  |  |  |
| C1QTNF6 |  |  |  | INHBA |  |  |  |  |  |
| C1QTNF7 |  |  |  | INHBB |  |  |  |  |  |
| C1RL |  |  |  | ISM1 |  |  |  |  |  |
| C1S |  |  |  | JAM3 |  |  |  |  |  |
| C2 |  |  |  | KERA |  |  |  |  |  |
| C2orf69 |  |  |  | LAMA4 |  |  |  |  |  |
| C3 |  |  |  | LCN6 |  |  |  |  |  |
| C4BPB |  |  |  | LGALS1 |  |  |  |  |  |
| C4orf48 |  |  |  | LILRA5 |  |  |  |  |  |
| CALCB |  |  |  | LOX |  |  |  |  |  |
| CBLN1 |  |  |  | LRRC17 |  |  |  |  |  |
| CBLN3 |  |  |  | LUM |  |  |  |  |  |
| CCBE1 |  |  |  | LUZP2 |  |  |  |  |  |
| CCDC126 |  |  |  | MDK |  |  |  |  |  |
| CCDC80 |  |  |  | MEP1B |  |  |  |  |  |
| CCL13 |  |  |  | METTL24 |  |  |  |  |  |
| CCL19 |  |  |  | MMP11 |  |  |  |  |  |
| CCL22 |  |  |  | MMP16 |  |  |  |  |  |
| CCL24 |  |  |  | MMP25 |  |  |  |  |  |
| CCL28 |  |  |  | MTRNR2L8 |  |  |  |  |  |
| CD14 |  |  |  | MZB1 |  |  |  |  |  |
| CD163 |  |  |  | NBL1 |  |  |  |  |  |
| CD40 |  |  |  | NHLRC3 |  |  |  |  |  |
| CD40LG |  |  |  | NPNT |  |  |  |  |  |
| CD44 |  |  |  | NPTX2 |  |  |  |  |  |
| CD55 |  |  |  | NRCAM |  |  |  |  |  |
| CDNF |  |  |  | OLFM1 |  |  |  |  |  |
| CDSN |  |  |  | OLFML2B |  |  |  |  |  |
| CETP |  |  |  | OLFML3 |  |  |  |  |  |
| CFB |  |  |  | PAPLN |  |  |  |  |  |
| CFD |  |  |  | PCOLCE |  |  |  |  |  |
| CFP |  |  |  | PCOLCE2 |  |  |  |  |  |
| CGREF1 |  |  |  | PCSK9 |  |  |  |  |  |
| CHIT1 |  |  |  | PDGFRL |  |  |  |  |  |
| CHL1 |  |  |  | PF4 |  |  |  |  |  |
| CHRDL1 |  |  |  | PGF |  |  |  |  |  |
| CHRDL2 |  |  |  | PI15 |  |  |  |  |  |
| CILP |  |  |  | PLA2G2A |  |  |  |  |  |
| CILP2 |  |  |  | PLA2G2C |  |  |  |  |  |
| CLCF1 |  |  |  | PLTP |  |  |  |  |  |
| CLEC18B |  |  |  | PODNL1 |  |  |  |  |  |
| CLEC18C |  |  |  | PPBP |  |  |  |  |  |
| CLEC3B |  |  |  | PRAP1 |  |  |  |  |  |
| CLU |  |  |  | PROK1 |  |  |  |  |  |
| CNDP1 |  |  |  | PROK2 |  |  |  |  |  |
| COCH |  |  |  | PROS1 |  |  |  |  |  |
| COL10A1 |  |  |  | PRSS23 |  |  |  |  |  |
| COL12A1 |  |  |  | PRXL2A |  |  |  |  |  |
| COL15A1 |  |  |  | PSG1 |  |  |  |  |  |
| COL16A1 |  |  |  | PSG11 |  |  |  |  |  |
| COL18A1 |  |  |  | PSG2 |  |  |  |  |  |
| COL19A1 |  |  |  | PSG3 |  |  |  |  |  |
| COL1A1 |  |  |  | PSG5 |  |  |  |  |  |
| COL1A2 |  |  |  | PSG6 |  |  |  |  |  |
| COL21A1 |  |  |  | PSG7 |  |  |  |  |  |
| COL24A1 |  |  |  | PSG8 |  |  |  |  |  |
| COL27A1 |  |  |  | PTGDS |  |  |  |  |  |
| COL28A1 |  |  |  | PTK7 |  |  |  |  |  |
| COL3A1 |  |  |  | PTX3 |  |  |  |  |  |
| COL4A1 |  |  |  | PXDN |  |  |  |  |  |
| COL4A2 |  |  |  | RARRES2 |  |  |  |  |  |
| COL4A3 |  |  |  | RBP4 |  |  |  |  |  |
| COL4A4 |  |  |  | RNASE4 |  |  |  |  |  |
| COL4A5 |  |  |  | S100A12 |  |  |  |  |  |
| COL4A6 |  |  |  | S100A8 |  |  |  |  |  |
| COL5A1 |  |  |  | S100A9 |  |  |  |  |  |
| COL5A2 |  |  |  | SCG2 |  |  |  |  |  |
| COL5A3 |  |  |  | SCN1B |  |  |  |  |  |
| COL6A1 |  |  |  | SCRG1 |  |  |  |  |  |
| COL6A2 |  |  |  | SDC1 |  |  |  |  |  |
| COL6A3 |  |  |  | SEMA3B |  |  |  |  |  |
| COL7A1 |  |  |  | SERPINA1 |  |  |  |  |  |
| COL9A1 |  |  |  | SERPING1 |  |  |  |  |  |
| COL9A3 |  |  |  | SFRP2 |  |  |  |  |  |
| COLQ |  |  |  | SFRP4 |  |  |  |  |  |
| COMP |  |  |  | SLPI |  |  |  |  |  |
| CPA2 |  |  |  | SMOC2 |  |  |  |  |  |
| CPA4 |  |  |  | SMPDL3A |  |  |  |  |  |
| CPB1 |  |  |  | SORL1 |  |  |  |  |  |
| CPN2 |  |  |  | SPARC |  |  |  |  |  |
| CPXM1 |  |  |  | SRPX2 |  |  |  |  |  |
| CPXM2 |  |  |  | SSC4D |  |  |  |  |  |
| CRB1 |  |  |  | ST6GAL1 |  |  |  |  |  |
| CRISP2 |  |  |  | TCN2 |  |  |  |  |  |
| CRISP3 |  |  |  | TFPI |  |  |  |  |  |
| CRISPLD1 |  |  |  | TFPI2 |  |  |  |  |  |
| CRLF1 |  |  |  | TGFB3 |  |  |  |  |  |
| CRTAC1 |  |  |  | TGFBI |  |  |  |  |  |
| CSF1 |  |  |  | THSD4 |  |  |  |  |  |
| CSF2RA |  |  |  | TIMP1 |  |  |  |  |  |
| CTF1 |  |  |  | TPSB2 |  |  |  |  |  |
| CTHRC1 |  |  |  | TUFT1 |  |  |  |  |  |
| CTRB1 |  |  |  | TWSG1 |  |  |  |  |  |
| CTRB2 |  |  |  | TXNDC16 |  |  |  |  |  |
| CTSB |  |  |  | UCN |  |  |  |  |  |
| CTSD |  |  |  | VCAN |  |  |  |  |  |
| CX3CL1 |  |  |  | VEGFB |  |  |  |  |  |
| CXADR |  |  |  | VMO1 |  |  |  |  |  |
| CXCL10 |  |  |  | ACP3 |  |  |  |  |  |
| CXCL11 |  |  |  | ADA2 |  |  |  |  |  |
| CXCL16 |  |  |  | ADAM28 |  |  |  |  |  |
| CXCL17 |  |  |  | ANGPTL8 |  |  |  |  |  |
| CXCL9 |  |  |  | APOL1 |  |  |  |  |  |
| DEFB1 |  |  |  | B2M |  |  |  |  |  |
| DEFB132 |  |  |  | C15orf61 |  |  |  |  |  |
| DHRSX |  |  |  | C5 |  |  |  |  |  |
| DIPK2A |  |  |  | CCDC134 |  |  |  |  |  |
| DKK2 |  |  |  | CCL14 |  |  |  |  |  |
| DMKN |  |  |  | CCL4L2 |  |  |  |  |  |
| DNASE1L2 |  |  |  | CCN5 |  |  |  |  |  |
| DPP4 |  |  |  | CLEC11A |  |  |  |  |  |
| DPP7 |  |  |  | COL11A1 |  |  |  |  |  |
| DPT |  |  |  | COL4A1 |  |  |  |  |  |
| ECM1 |  |  |  | COL4A2 |  |  |  |  |  |
| EDDM13 |  |  |  | COLEC11 |  |  |  |  |  |
| EDN2 |  |  |  | CUTA |  |  |  |  |  |
| EFEMP1 |  |  |  | CXADR |  |  |  |  |  |
| EFNA1 |  |  |  | EBI3 |  |  |  |  |  |
| EGFL6 |  |  |  | ENTPD5 |  |  |  |  |  |
| EGFL7 |  |  |  | GASK1A |  |  |  |  |  |
| EGFR |  |  |  | GDF1 |  |  |  |  |  |
| ELN |  |  |  | GLDN |  |  |  |  |  |
| EMC10 |  |  |  | GZMH |  |  |  |  |  |
| EMID1 |  |  |  | HLA-E |  |  |  |  |  |
| EMILIN1 |  |  |  | HLA-G |  |  |  |  |  |
| EMILIN2 |  |  |  | HPR |  |  |  |  |  |
| EMILIN3 |  |  |  | IGIP |  |  |  |  |  |
| ENAM |  |  |  | IGLON5 |  |  |  |  |  |
| ENDOD1 |  |  |  | IL15RA |  |  |  |  |  |
| ENPP1 |  |  |  | IL16 |  |  |  |  |  |
| ENPP2 |  |  |  | IL1RAP |  |  |  |  |  |
| EPDR1 |  |  |  | KIAA0100 |  |  |  |  |  |
| ERAP1 |  |  |  | KRT10 |  |  |  |  |  |
| ERAP2 |  |  |  | LOXL2 |  |  |  |  |  |
| ERBB3 |  |  |  | LPL |  |  |  |  |  |
| ERFE |  |  |  | LRG1 |  |  |  |  |  |
| F13A1 |  |  |  | LY86 |  |  |  |  |  |
| F3 |  |  |  | LY96 |  |  |  |  |  |
| FBLN2 |  |  |  | MANF |  |  |  |  |  |
| FBLN5 |  |  |  | MATN2 |  |  |  |  |  |
| FBLN7 |  |  |  | MATN3 |  |  |  |  |  |
| FBN1 |  |  |  | MGAT4A |  |  |  |  |  |
| FCER2 |  |  |  | MMRN1 |  |  |  |  |  |
| FCGBP |  |  |  | NUCB2 |  |  |  |  |  |
| FCGR3A |  |  |  | ORM2 |  |  |  |  |  |
| FCMR |  |  |  | PDCD1LG2 |  |  |  |  |  |
| FCN1 |  |  |  | PEBP4 |  |  |  |  |  |
| FCN2 |  |  |  | PINLYP |  |  |  |  |  |
| FCN3 |  |  |  | PON1 |  |  |  |  |  |
| FGF10 |  |  |  | PTPRS |  |  |  |  |  |
| FGF17 |  |  |  | PTX4 |  |  |  |  |  |
| FGF21 |  |  |  | S100A13 |  |  |  |  |  |
| FKRP |  |  |  | SCPEP1 |  |  |  |  |  |
| FLT4 |  |  |  | SCT |  |  |  |  |  |
| FMOD |  |  |  | SEMA3G |  |  |  |  |  |
| FN1 |  |  |  | SERPINB1 |  |  |  |  |  |
| FOLR1 |  |  |  | SMPDL3B |  |  |  |  |  |
| FOLR2 |  |  |  | SOD3 |  |  |  |  |  |
| FRZB |  |  |  | TIMP4 |  |  |  |  |  |
| FST |  |  |  | TNFRSF18 |  |  |  |  |  |
| FSTL1 |  |  |  | UTS2B |  |  |  |  |  |
| FSTL3 |  |  |  | VASH2 |  |  |  |  |  |
| FTCDNL1 |  |  |  | VSTM2L |  |  |  |  |  |
| FUCA2 |  |  |  | WNT11 |  |  |  |  |  |
| GAL |  |  |  | WNT8B |  |  |  |  |  |
| GAS6 |  |  |  | XCL1 |  |  |  |  |  |
| GDF1 |  |  |  | ZG16B |  |  |  |  |  |
| GDF10 |  |  |  |  |  |  |  |  |  |
| GDF5 |  |  |  |  |  |  |  |  |  |
| GDF7 |  |  |  |  |  |  |  |  |  |
| GFOD2 |  |  |  |  |  |  |  |  |  |
| GHR |  |  |  |  |  |  |  |  |  |
| GNLY |  |  |  |  |  |  |  |  |  |
| GNRH2 |  |  |  |  |  |  |  |  |  |
| GPLD1 |  |  |  |  |  |  |  |  |  |
| GREM1 |  |  |  |  |  |  |  |  |  |
| GREM2 |  |  |  |  |  |  |  |  |  |
| GRN |  |  |  |  |  |  |  |  |  |
| GZMB |  |  |  |  |  |  |  |  |  |
| HAPLN1 |  |  |  |  |  |  |  |  |  |
| HDGF |  |  |  |  |  |  |  |  |  |
| HGFAC |  |  |  |  |  |  |  |  |  |
| HLA-E |  |  |  |  |  |  |  |  |  |
| HLA-G |  |  |  |  |  |  |  |  |  |
| HMGB2 |  |  |  |  |  |  |  |  |  |
| HPSE |  |  |  |  |  |  |  |  |  |
| HSPG2 |  |  |  |  |  |  |  |  |  |
| HTRA1 |  |  |  |  |  |  |  |  |  |
| HTRA3 |  |  |  |  |  |  |  |  |  |
| IFI35 |  |  |  |  |  |  |  |  |  |
| IFNAR2 |  |  |  |  |  |  |  |  |  |
| IFNK |  |  |  |  |  |  |  |  |  |
| IGFALS |  |  |  |  |  |  |  |  |  |
| IGSF10 |  |  |  |  |  |  |  |  |  |
| IL10 |  |  |  |  |  |  |  |  |  |
| IL15RA |  |  |  |  |  |  |  |  |  |
| IL17RA |  |  |  |  |  |  |  |  |  |
| IL1R1 |  |  |  |  |  |  |  |  |  |
| IL1RAP |  |  |  |  |  |  |  |  |  |
| IL4R |  |  |  |  |  |  |  |  |  |
| IL6R |  |  |  |  |  |  |  |  |  |
| IL6ST |  |  |  |  |  |  |  |  |  |
| IMPG1 |  |  |  |  |  |  |  |  |  |
| INHBB |  |  |  |  |  |  |  |  |  |
| ISG15 |  |  |  |  |  |  |  |  |  |
| ITGBL1 |  |  |  |  |  |  |  |  |  |
| ITIH4 |  |  |  |  |  |  |  |  |  |
| ITIH5 |  |  |  |  |  |  |  |  |  |
| ITIH6 |  |  |  |  |  |  |  |  |  |
| ITM2B |  |  |  |  |  |  |  |  |  |
| KAZALD1 |  |  |  |  |  |  |  |  |  |
| KERA |  |  |  |  |  |  |  |  |  |
| KIAA0100 |  |  |  |  |  |  |  |  |  |
| LAD1 |  |  |  |  |  |  |  |  |  |
| LAIR2 |  |  |  |  |  |  |  |  |  |
| LAMA4 |  |  |  |  |  |  |  |  |  |
| LAMA5 |  |  |  |  |  |  |  |  |  |
| LAMB2 |  |  |  |  |  |  |  |  |  |
| LAMB3 |  |  |  |  |  |  |  |  |  |
| LAMC1 |  |  |  |  |  |  |  |  |  |
| LAMC2 |  |  |  |  |  |  |  |  |  |
| LBP |  |  |  |  |  |  |  |  |  |
| LCN15 |  |  |  |  |  |  |  |  |  |
| LCN8 |  |  |  |  |  |  |  |  |  |
| LDLRAD2 |  |  |  |  |  |  |  |  |  |
| LEFTY2 |  |  |  |  |  |  |  |  |  |
| LGALS3 |  |  |  |  |  |  |  |  |  |
| LGALS3BP |  |  |  |  |  |  |  |  |  |
| LGALS9 |  |  |  |  |  |  |  |  |  |
| LGI4 |  |  |  |  |  |  |  |  |  |
| LILRA5 |  |  |  |  |  |  |  |  |  |
| LIPC |  |  |  |  |  |  |  |  |  |
| LIPF |  |  |  |  |  |  |  |  |  |
| LOX |  |  |  |  |  |  |  |  |  |
| LOXL2 |  |  |  |  |  |  |  |  |  |
| LPL |  |  |  |  |  |  |  |  |  |
| LRP8 |  |  |  |  |  |  |  |  |  |
| LTBP1 |  |  |  |  |  |  |  |  |  |
| LTBP3 |  |  |  |  |  |  |  |  |  |
| LUM |  |  |  |  |  |  |  |  |  |
| LUZP2 |  |  |  |  |  |  |  |  |  |
| LY6G5C |  |  |  |  |  |  |  |  |  |
| LY86 |  |  |  |  |  |  |  |  |  |
| LY96 |  |  |  |  |  |  |  |  |  |
| LYG1 |  |  |  |  |  |  |  |  |  |
| LYG2 |  |  |  |  |  |  |  |  |  |
| LYZ |  |  |  |  |  |  |  |  |  |
| MAN2B2 |  |  |  |  |  |  |  |  |  |
| MAPT |  |  |  |  |  |  |  |  |  |
| MATN3 |  |  |  |  |  |  |  |  |  |
| MDK |  |  |  |  |  |  |  |  |  |
| MEGF6 |  |  |  |  |  |  |  |  |  |
| MET |  |  |  |  |  |  |  |  |  |
| METRNL |  |  |  |  |  |  |  |  |  |
| MFAP2 |  |  |  |  |  |  |  |  |  |
| MGAT4A |  |  |  |  |  |  |  |  |  |
| MMP12 |  |  |  |  |  |  |  |  |  |
| MMP17 |  |  |  |  |  |  |  |  |  |
| MMP19 |  |  |  |  |  |  |  |  |  |
| MMP2 |  |  |  |  |  |  |  |  |  |
| MMP21 |  |  |  |  |  |  |  |  |  |
| MMP24 |  |  |  |  |  |  |  |  |  |
| MMP9 |  |  |  |  |  |  |  |  |  |
| MMRN1 |  |  |  |  |  |  |  |  |  |
| MPEG1 |  |  |  |  |  |  |  |  |  |
| MTRNR2L8 |  |  |  |  |  |  |  |  |  |
| MUC1 |  |  |  |  |  |  |  |  |  |
| MUC4 |  |  |  |  |  |  |  |  |  |
| MUC6 |  |  |  |  |  |  |  |  |  |
| MXRA7 |  |  |  |  |  |  |  |  |  |
| MYDGF |  |  |  |  |  |  |  |  |  |
| MYOC |  |  |  |  |  |  |  |  |  |
| MZB1 |  |  |  |  |  |  |  |  |  |
| NCAM1 |  |  |  |  |  |  |  |  |  |
| NCAN |  |  |  |  |  |  |  |  |  |
| NDNF |  |  |  |  |  |  |  |  |  |
| NECTIN1 |  |  |  |  |  |  |  |  |  |
| NELL1 |  |  |  |  |  |  |  |  |  |
| NHLRC3 |  |  |  |  |  |  |  |  |  |
| NID1 |  |  |  |  |  |  |  |  |  |
| NMB |  |  |  |  |  |  |  |  |  |
| NMI |  |  |  |  |  |  |  |  |  |
| NOG |  |  |  |  |  |  |  |  |  |
| NOTUM |  |  |  |  |  |  |  |  |  |
| NPC2 |  |  |  |  |  |  |  |  |  |
| NPTX2 |  |  |  |  |  |  |  |  |  |
| NPY |  |  |  |  |  |  |  |  |  |
| NRCAM |  |  |  |  |  |  |  |  |  |
| NRG2 |  |  |  |  |  |  |  |  |  |
| NRP1 |  |  |  |  |  |  |  |  |  |
| NRP2 |  |  |  |  |  |  |  |  |  |
| NTF4 |  |  |  |  |  |  |  |  |  |
| NTN4 |  |  |  |  |  |  |  |  |  |
| NUCB1 |  |  |  |  |  |  |  |  |  |
| NXPE3 |  |  |  |  |  |  |  |  |  |
| NXPH4 |  |  |  |  |  |  |  |  |  |
| OAF |  |  |  |  |  |  |  |  |  |
| OIT3 |  |  |  |  |  |  |  |  |  |
| OLFM2 |  |  |  |  |  |  |  |  |  |
| OLFM4 |  |  |  |  |  |  |  |  |  |
| OLFML2B |  |  |  |  |  |  |  |  |  |
| OSTN |  |  |  |  |  |  |  |  |  |
| OTOGL |  |  |  |  |  |  |  |  |  |
| OVCH2 |  |  |  |  |  |  |  |  |  |
| OXT |  |  |  |  |  |  |  |  |  |
| PAM |  |  |  |  |  |  |  |  |  |
| PAMR1 |  |  |  |  |  |  |  |  |  |
| PAPLN |  |  |  |  |  |  |  |  |  |
| PCDH12 |  |  |  |  |  |  |  |  |  |
| PCOLCE |  |  |  |  |  |  |  |  |  |
| PCOLCE2 |  |  |  |  |  |  |  |  |  |
| PCSK6 |  |  |  |  |  |  |  |  |  |
| PDCD1LG2 |  |  |  |  |  |  |  |  |  |
| PDGFA |  |  |  |  |  |  |  |  |  |
| PDGFC |  |  |  |  |  |  |  |  |  |
| PEBP4 |  |  |  |  |  |  |  |  |  |
| PILRA |  |  |  |  |  |  |  |  |  |
| PKDCC |  |  |  |  |  |  |  |  |  |
| PLA2G2D |  |  |  |  |  |  |  |  |  |
| PLA2G7 |  |  |  |  |  |  |  |  |  |
| PLA2R1 |  |  |  |  |  |  |  |  |  |
| PLAT |  |  |  |  |  |  |  |  |  |
| PLOD3 |  |  |  |  |  |  |  |  |  |
| PLTP |  |  |  |  |  |  |  |  |  |
| PNLIPRP3 |  |  |  |  |  |  |  |  |  |
| PON2 |  |  |  |  |  |  |  |  |  |
| POSTN |  |  |  |  |  |  |  |  |  |
| PPIB |  |  |  |  |  |  |  |  |  |
| PRADC1 |  |  |  |  |  |  |  |  |  |
| PRB3 |  |  |  |  |  |  |  |  |  |
| PROS1 |  |  |  |  |  |  |  |  |  |
| PRSS16 |  |  |  |  |  |  |  |  |  |
| PRSS23 |  |  |  |  |  |  |  |  |  |
| PRSS36 |  |  |  |  |  |  |  |  |  |
| PRXL2A |  |  |  |  |  |  |  |  |  |
| PSG1 |  |  |  |  |  |  |  |  |  |
| PSG11 |  |  |  |  |  |  |  |  |  |
| PSG3 |  |  |  |  |  |  |  |  |  |
| PSG5 |  |  |  |  |  |  |  |  |  |
| PSG6 |  |  |  |  |  |  |  |  |  |
| PSG7 |  |  |  |  |  |  |  |  |  |
| PSG8 |  |  |  |  |  |  |  |  |  |
| PTK7 |  |  |  |  |  |  |  |  |  |
| PTN |  |  |  |  |  |  |  |  |  |
| PTPRS |  |  |  |  |  |  |  |  |  |
| PTX3 |  |  |  |  |  |  |  |  |  |
| PXDN |  |  |  |  |  |  |  |  |  |
| QPCT |  |  |  |  |  |  |  |  |  |
| QSOX1 |  |  |  |  |  |  |  |  |  |
| RARRES2 |  |  |  |  |  |  |  |  |  |
| RBP4 |  |  |  |  |  |  |  |  |  |
| RGMA |  |  |  |  |  |  |  |  |  |
| RNASE7 |  |  |  |  |  |  |  |  |  |
| RNASET2 |  |  |  |  |  |  |  |  |  |
| RNLS |  |  |  |  |  |  |  |  |  |
| RNPEP |  |  |  |  |  |  |  |  |  |
| RSPO1 |  |  |  |  |  |  |  |  |  |
| RSPO3 |  |  |  |  |  |  |  |  |  |
| SAA1 |  |  |  |  |  |  |  |  |  |
| SAA2 |  |  |  |  |  |  |  |  |  |
| SCG2 |  |  |  |  |  |  |  |  |  |
| SCG5 |  |  |  |  |  |  |  |  |  |
| SCGB3A1 |  |  |  |  |  |  |  |  |  |
| SCN1B |  |  |  |  |  |  |  |  |  |
| SCT |  |  |  |  |  |  |  |  |  |
| SCUBE2 |  |  |  |  |  |  |  |  |  |
| SDC1 |  |  |  |  |  |  |  |  |  |
| SECTM1 |  |  |  |  |  |  |  |  |  |
| SEMA3A |  |  |  |  |  |  |  |  |  |
| SEMA3C |  |  |  |  |  |  |  |  |  |
| SEMA3D |  |  |  |  |  |  |  |  |  |
| SEMA3G |  |  |  |  |  |  |  |  |  |
| SERPIND1 |  |  |  |  |  |  |  |  |  |
| SERPINE1 |  |  |  |  |  |  |  |  |  |
| SERPING1 |  |  |  |  |  |  |  |  |  |
| SFTPD |  |  |  |  |  |  |  |  |  |
| SLIT3 |  |  |  |  |  |  |  |  |  |
| SLPI |  |  |  |  |  |  |  |  |  |
| SMOC1 |  |  |  |  |  |  |  |  |  |
| SNED1 |  |  |  |  |  |  |  |  |  |
| SOD3 |  |  |  |  |  |  |  |  |  |
| SOGA1 |  |  |  |  |  |  |  |  |  |
| SPARC |  |  |  |  |  |  |  |  |  |
| SPATA20 |  |  |  |  |  |  |  |  |  |
| SPESP1 |  |  |  |  |  |  |  |  |  |
| SPINK13 |  |  |  |  |  |  |  |  |  |
| SPINK5 |  |  |  |  |  |  |  |  |  |
| SPON1 |  |  |  |  |  |  |  |  |  |
| SPON2 |  |  |  |  |  |  |  |  |  |
| SPP1 |  |  |  |  |  |  |  |  |  |
| SRGN |  |  |  |  |  |  |  |  |  |
| SRPX |  |  |  |  |  |  |  |  |  |
| SRPX2 |  |  |  |  |  |  |  |  |  |
| STC2 |  |  |  |  |  |  |  |  |  |
| SVBP |  |  |  |  |  |  |  |  |  |
| TECTA |  |  |  |  |  |  |  |  |  |
| TF |  |  |  |  |  |  |  |  |  |
| TFPI2 |  |  |  |  |  |  |  |  |  |
| TGFB3 |  |  |  |  |  |  |  |  |  |
| TGFBI |  |  |  |  |  |  |  |  |  |
| TGFBR3 |  |  |  |  |  |  |  |  |  |
| THBS1 |  |  |  |  |  |  |  |  |  |
| THBS2 |  |  |  |  |  |  |  |  |  |
| THBS4 |  |  |  |  |  |  |  |  |  |
| THEM6 |  |  |  |  |  |  |  |  |  |
| TIMP1 |  |  |  |  |  |  |  |  |  |
| TIMP2 |  |  |  |  |  |  |  |  |  |
| TIMP3 |  |  |  |  |  |  |  |  |  |
| TINAG |  |  |  |  |  |  |  |  |  |
| TLL2 |  |  |  |  |  |  |  |  |  |
| TNC |  |  |  |  |  |  |  |  |  |
| TNFRSF18 |  |  |  |  |  |  |  |  |  |
| TNFRSF1A |  |  |  |  |  |  |  |  |  |
| TNFRSF1B |  |  |  |  |  |  |  |  |  |
| TNFRSF25 |  |  |  |  |  |  |  |  |  |
| TNFSF15 |  |  |  |  |  |  |  |  |  |
| TNN |  |  |  |  |  |  |  |  |  |
| TNR |  |  |  |  |  |  |  |  |  |
| TNXB |  |  |  |  |  |  |  |  |  |
| TREM2 |  |  |  |  |  |  |  |  |  |
| TRH |  |  |  |  |  |  |  |  |  |
| TUFT1 |  |  |  |  |  |  |  |  |  |
| TULP2 |  |  |  |  |  |  |  |  |  |
| UCN |  |  |  |  |  |  |  |  |  |
| VASH1 |  |  |  |  |  |  |  |  |  |
| VASN |  |  |  |  |  |  |  |  |  |
| VEGFA |  |  |  |  |  |  |  |  |  |
| VEGFB |  |  |  |  |  |  |  |  |  |
| VEGFC |  |  |  |  |  |  |  |  |  |
| VEGFD |  |  |  |  |  |  |  |  |  |
| VIT |  |  |  |  |  |  |  |  |  |
| VWA1 |  |  |  |  |  |  |  |  |  |
| VWA7 |  |  |  |  |  |  |  |  |  |
| VWCE |  |  |  |  |  |  |  |  |  |
| VWDE |  |  |  |  |  |  |  |  |  |
| WFDC2 |  |  |  |  |  |  |  |  |  |
| WFIKKN1 |  |  |  |  |  |  |  |  |  |
| WFIKKN2 |  |  |  |  |  |  |  |  |  |
| WIF1 |  |  |  |  |  |  |  |  |  |
| WNT16 |  |  |  |  |  |  |  |  |  |
| WNT4 |  |  |  |  |  |  |  |  |  |
| WNT5A |  |  |  |  |  |  |  |  |  |
| WNT7B |  |  |  |  |  |  |  |  |  |
| ZBED3 |  |  |  |  |  |  |  |  |  |
| ZG16B |  |  |  |  |  |  |  |  |  |
| ZP1 |  |  |  |  |  |  |  |  |  |

**References**

1. Meyer GA, Gibbons MC, Sato E, Lane JG, Ward SR, Engler AJ. Epimuscular Fat in the Human Rotator Cuff Is a Novel Beige Depot. *Stem Cells Transl Med*. 2015;4(7):764-774. doi:10.5966/sctm.2014-0287

2. Zuk PA, Zhu M, Ashjian P, et al. Human adipose tissue is a source of multipotent stem cells. *Mol Biol Cell*. 2002;13(12):4279-4295. doi:10.1091/mbc.e02-02-0105

3. Bohnert KL, Hastings MK, Sinacore DR, et al. Skeletal Muscle Regeneration in Advanced Diabetic Peripheral Neuropathy. *Foot Ankle Int*. 2020;41(5):536-548. doi:10.1177/1071100720907035
